# Breakfast: A Crucial Meal for Adolescents’ Cognitive Performance According to Their Nutritional Status. The Cogni-Action Project

**DOI:** 10.3390/nu13041320

**Published:** 2021-04-16

**Authors:** Humberto Peña-Jorquera, Valentina Campos-Núñez, Kabir P. Sadarangani, Gerson Ferrari, Carlos Jorquera-Aguilera, Carlos Cristi-Montero

**Affiliations:** 1 IRyS Group, Physical Education School, Pontificia Universidad Católica de Valparaíso, Viña del Mar 2530388, Chile; humberto.apj@gmail.com (H.P.-J.); camposnunezvalentina@gmail.com (V.C.-N.); 2 Universidad Autónoma de Chile, Providencia 7500912, Chile; kabir.sadarangani@gmail.com; 3 Escuela de Kinesiología, Facultad de Salud y Odontología, Universidad Diego Portales, Santiago 8370057, Chile; 4 Escuela de Ciencias de la Actividad Física, el Deporte y la Salud, Universidad de Santiago de Chile (USACH), Santiago 7500618, Chile; gersonferrari08@yahoo.com.br; 5 Escuela de Nutrición y Dietética, Facultad de Ciencias, Universidad Mayor, Santiago 8580745, Chile; carlos.jorquera@mayor.cl

**Keywords:** breakfast, cognition, children, obesity, meals, nutrition, breakfast skippers

## Abstract

This study aimed to determine whether pupils who have breakfast just before a cognitive demand, do not regularly skip breakfast, and consume a high-quality breakfast present higher cognitive performance than those who do not; furthermore, to establish differences according to their nutritional status. In this study, 1181 Chilean adolescents aged 10–14 years participated. A global cognitive score was computed through eight tasks, and the body mass index z-score (BMIz) was calculated using a growth reference for school-aged adolescents. The characteristics of breakfast were self-reported. Analyses of covariance were performed to determine differences in cognitive performance according to BMIz groups adjusted to sex, peak height velocity, physical fitness global score, and their schools. A positive association was found in adolescents’ cognitive performance when they had breakfast just before cognitive tasks, did not regularly skip breakfast, presented at least two breakfast quality components, and included dairy products. No significant differences were found between breakfast components, including cereal/bread and fruits/fruit juice. Finally, pupils who were overweight/obese who declared that they skipped breakfast regularly presented a lower cognitive performance than their normal-BMIz peers. These findings suggest that adolescents who have breakfast just prior to a cognitive demand and regularly have a high quality breakfast have better cognitive performance than those who do not. Educative nutritional strategies should be prioritized, especially in “breakfast skippers” adolescents living with overweight/obesity.

## 1. Background

Complete and balanced nutrition is essential to maintain health in optimal conditions and avoid health risks throughout the entire life cycle. However, during the development stage and, particularly, during early adolescence, nutrition becomes essential due to its influences on brain maturation and future health indicators [1,2]. It is well-known that, in childhood and adolescence, food habits are strongly influenced by family, guardians, culture, and socio-economic context, which impact meals’ frequency and quality [3,4]. Therefore, adolescents’ eating patterns play a fundamental role in their global health, behavior, and cognitive functioning, which are relevant outcomes at the scholar age [2].

Breakfast has been recognized as an essential meal associated with high intake of proteins, vitamins, and minerals, and raised glucose blood concentration [5,6,7]. Moreover, a high-quality breakfast has been related to a lower risk of obesity [4]. Thereby, both a nutrient deficit and obesity have been linked to lower cognitive functioning during childhood [8,9,10]. Having a regular breakfast and improving its quality could enhance children’s cognitive performance [11,12] through enhanced episodic and visual memory, attention, and other cognitive skills [13,14,15]. For instance, a large-scale and empirical study of Chinese elementary and middle school students’ data (*N* = 56,238 and *N* = 91,543, respectively) concluded that eating breakfast every day had a significant effect on academic quality (a proxy variable for cognitive development) [12].

However, despite the documented benefits of breakfast consumption, skipping breakfast is more common than specialists expect. Depending on the definition of skipping breakfast and the studied age group, the prevalence ranges from 10% to 74.7% in children and adolescents [16,17]. Notably, the frequency of skipping breakfast increases in adolescents [16,18] and is higher in girls [16,19], and “breakfast skippers” tend to consume more fast food, leading to increased weight gain from adolescence to adulthood [20]. Similarly, adolescents with an excess of adiposity have decreased cognitive functioning [8,9].

The fundamentals underlying the interactions among obesity with brain health and cognition involve neuroelectric indicators [21], the energy demand of the brain [22], low-grade inflammation and hormonal production alteration [22], grey and white brain matter volumes [23], and phagocytosis of synapses by microglia [24], among others. This complex and detrimental environment converges on a reduction in brain activity, affecting cognitive performance. However, currently, the literature is inconsistent regarding how nutritional status influences cognitive functioning in childhood [25]; thus, a challenge in this area is establishing the role of breakfast on this relationship (obesity/cognition) [4].

This study seeks to elucidate the relationship between breakfast and cognition according to the adolescents’ nutritional status, covering a geographical gap. Most studies on this subject come from developed countries with more favorable economic and social contexts. Therefore, based on a large sample of Chilean pupils, the primary study aim was to determine whether those who have breakfast just before a complete cognitive battery, do not regularly skip breakfast, and consume a high-quality breakfast present higher cognitive performance than those who do not. Moreover, the purpose was to establish differences according to their nutritional status.

## 2. Materials and Methods

### 2.1. Study Design

This study is part of the Cogni-Action Project, which seeks to establish the association among physical activity, sedentary behavior, and physical fitness with brain structure and function, cognitive performance, and academic achievement in Chilean adolescents [26]. More information on the Cogni-Action project is provided elsewhere [26]. This project was retrospectively registered (8 July 2020) in the Research Registry (ID: researchregistry5791). The cross-sectional project was carried out from March 2017 to October 2019, where 10–14 years-old (5th to 8th grades) adolescents were recruited from the public, voucher, and private schools in Valparaiso, Chile.

### 2.2. Study Population

Total sample size and power calculations were based on pupils’ total enrolment from 5th to 8th grades in the Valparaiso region indicated by the Chilean Ministry of Education in the year 2016 (universe *n* = 951,962). It was based on a proportion and considered to have a heterogeneity of 50%, confidence interval of 99%, 5% error range, and a dropout of 20%. Thus, a total of 797 participants were necessary to have a representative sample. The general inclusion criteria were a girls and boys from 5th to 8th grades. A total of 1586 pupils were involved in this project. For the present study, 1181 pupils were included after applying the exclusion criteria (a) being out of the age range, and (b) not participating in the cognitive evaluation.

### 2.3. Measurements

Measurements took place in schools between 9:00 and 15:00 through two sessions of four hours each, separated by eight days apart. In the early hours of the morning (session one), a complete cognitive battery was applied, and after that, anthropometric measurements and nutritional questionnaires were evaluated. In session two, physical fitness was assessed. Trained instructors from our research team guided all evaluations, and pupils had a brief familiarization trial before each test.

#### 2.3.1. Cognitive Performance

Adolescents’ cognitive performance was assessed through the NeuroCognitive Performance Test (NCPT) from Lumos Labs, Inc., San Francisco, CA, USA. The NCPT is a brief, modular, and online neuropsychological assessment platform [27]. It was applied in schoolrooms, in groups of 25 pupils, each with a laptop connected to the internet. The entire session lasted around one hour, which consisted of a brief explanation about the session’s aim, a demonstration, practice before each task, and the execution.

The NCPT is made up of 17 subtasks that each researcher can combine to build custom test batteries. In this sense, we selected eight tasks to evaluate several cognitive skills, such as working memory, processing speed, problem-solving, selective and divided attention, fluid and logical reasoning, and cognitive flexibility (details in Solis-Urra et al., 2019) [26]. A global cognitive performance score was computed; thus, each test was scaled following a normal inverse transformation of the percentile rank 25. These procedures benefitted from having scaled scores derived on the same normal distribution with a mean of 100 and a standard deviation of 15. Therefore, a mean of the eight tasks was calculated and used as an indicator of global cognitive performance.

#### 2.3.2. Nutritional Status

Pupils wore shorts, a t-shirt, and no trainers when their body weight (kg) and height (cm) were measured. A digital balance was used for measuring weight with precision between 0.1 kg and 150 kg (OMROM, HN-289-LA, Kyoto, Japan), while height was measured using a portable stadiometer (SECA, model 213, GmbH Co., Hamburg, Germany). A nutritional status indicator, the body mass index z-score (BMIz), was determined using the World Health Organization (WHO) 2007 growth reference for children and adolescents adjusted to their age and sex [28]. This indicator considers that a child’s BMIz cannot be fixed as in adults because their fat levels changes over time (age) and mature (growth patterns differ between boys and girls). According to WHO recommendations for children and adolescents ranged 5–19 years, the thresholds were as follow: thinness: <−2SD, normal: between >−2SD to <+1SD, overweight: between +1SD and <+2SD, and obesity: >+2SD. In this study, two categories were computed: “normal-BMIz” and adolescents with “overweight/obesity”. Only three cases were <−2SD and were excluded from the analysis.

#### 2.3.3. Breakfast Information

Several nutritional questions and a validated questionnaire (self-reported) were applied during the first evaluation session. Just before pupils took their cognitive performance test, we asked whether they had breakfast (yes/no). Based on a question from the Mediterranean Diet Quality Index (KIDMED) for children and adolescents [29], we asked whether they skipped breakfast regularly (yes/no). Additionally, a breakfast quality score was computed according to the EndKid Study criteria [30], assessing for (yes/no) a portion of (a) cereals/bread, (b) dairy (e.g., milk, yoghurt, cheese), and (c) fruits or natural juice without sugar in their breakfast. Thus, 1 point was awarded for each of the three food groups, resulting in a possible score of 0 to 3.

#### 2.3.4. Covariates

Sex, peak high velocity (PHV), physical fitness, and schools were used as covariates in all models. The BMIz-score was added to the model when the analysis involved all adolescents without splitting them by nutritional status. Sex, age, and maturation are personal modulators of cognitive development [31]. Thus, PHV was considered a maturity status indicator computed from age and height following Moore’s equations [32]. The PHV age was subtracted from the chronological age, and the difference in years was defined as the value of maturity offset, permitting for comparisons of somatic maturity between boys and girls. BMIz and physical fitness are critical behavioral moderators of adolescents’ cognitive performance [26]. BMIz measurement was described previously. A global score of physical fitness was computed adding z-scores based on sex and age as a normalized score of muscular, cardiorespiratory, and speed-agility fitness. Overall, physical fitness was evaluated by the ALPHA-fitness test battery [33]. This is a valid, reliable, feasible, and safe field-based fitness test in children and adolescents that permits the assessment and monitoring of many pupils simultaneously. All tests were performed in sports fields or an indoor gym during the morning, and pupils wore appropriate sportswear. Adolescents practised each test previously guided by a trainer and then started when they felt confident. Finally, school was included as a covariate due to the association between cognitive performance, socio-economic background, physical fitness, and academic performance [34,35]. In children from Latin-America, cognitive and academic achievements seem to be more closely related to school characteristics (i.e., economic, social, and cultural status) than variables associated with socioeconomic status (personal/familiar), which would explain to a great extent the outcome of the present study (cognitive performance) [36].

#### 2.3.5. Statistical Analyses

Descriptive and comparative analyses are presented as means (and their standard deviation) and frequency (and their percentage). Differences between BMIz groups in continuous and factor variables were tested using the Student’s *t*-test for equal variances and the Chi-square test, respectively (Table 1). Parametric analyses were carried out according to the central limit theorem, which indicates that, when the sample size is over 500, parametric methods are safe even though skewed data are present [37]. However, simultaneously, Q-Q visual analysis was performed, and Leven’s test range from *p* = 390 to *p* = 0.740. One-way analyses of covariance (ANCOVA) were performed to assess the mean differences in adolescents’ cognitive performance (adjusted by sex, PHV, BMIz, and schools) and according to BMIz groups (adjusted by sex, PHV, and schools). A Tukey post-hoc pairwise comparison was conducted to establish differences in the marginal estimated means in each pair of groups. Additionally, two effect size estimations were used, the first (η^2^p) for the global analysis and the second (Cohen’s d) for comparisons between groups. The η^2^p was interpreted as small at 0.01, medium at 0.06, and large at 0.14; while the Cohen’s d was interpreted as no effect (<0.2), small effect (0.2 < 0.5), medium effect (0.5 < 0.8), and large effect (≥0.8) [38]. The significance level was set at *p* < 0.05. All statistical analyses were performed with the free and open statistical software JAMOVI [Computer Software] (version 1.6.7.0, Sydney, Australia).

## 3. Results

Table 1 shows the participant characteristics of the study. The sample consisted of 50.9% boys, and 52.3% of pupils presented overweight or obesity. Moreover, 24% of adolescents declared themselves “breakfast skippers”. Significant differences were found between BMIz groups in several personal characteristics and the primary outcomes, such as BMIz and cognitive performance. There were no significant differences in most breakfast variables, only in the skipping breakfast regularly item.

**Figure 1:**
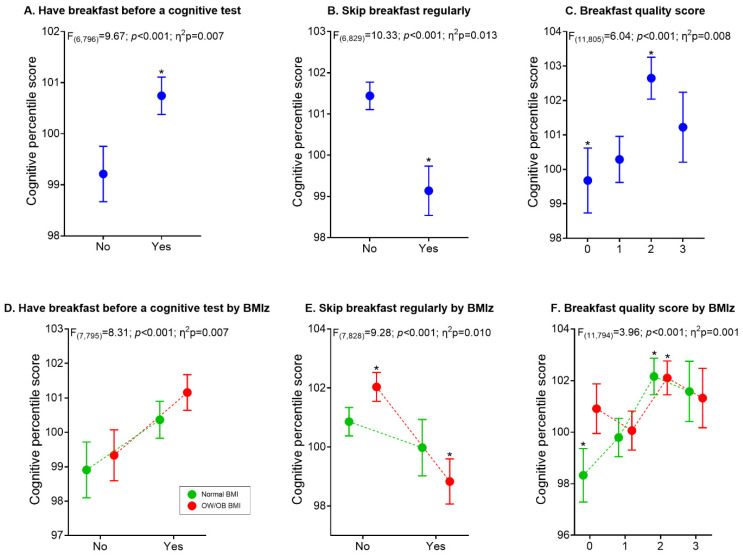
Differences in adolescents’ cognitive performance according to breakfast consumption and quality. Models in figures (**A–C**) were adjusted to sex, peak high velocity, physical fitness, schools, and body mass index z-score. In contrast, models in figures (**D**–**F**) were adjusted to sex, peak high velocity, fitness, and schools. * Significative mean differences between groups. BMI: body mass index; OW: overweight; OB; obesity.

Figure 1 shows the results of the three main breakfast variables associated with cognitive performance in all adolescents (Figure 1A–C) and according to their nutritional status (Figure 1D–F). All six general models were statistically significant (*p* < 0.001). Comparative analyses showed that (A) adolescents who had breakfast just before a cognitive test presented significant differences in comparison to those who did not (*p* = 0.020; Cohen’s d = 0.18); (B) pupils who skipped breakfast regularly underperformed in the cognitive test (*p* = 0.001; Cohen’s d = 0.27); and (C) higher cognitive performance was observed when at least two components were included in breakfast (0 vs. 2 components: *p* = 0.001; Cohen’s d = 0.30). Analyses according to BMIz groups showed that (D) no differences were found between groups regarding who had breakfast just before a cognitive test or not; (E) a significant and negative difference was observed in adolescents with overweight/obesity who skipped breakfast regularly (*p* = 0.002; Cohen’s d = 0.38); and (F) regarding breakfast quality, differences were observed in normal-BMIz pupils (0 vs. 2 components; *p* = 0.053; Cohen’s d = 0.45) and the same group (normal-BMIz) compared to the overweight/obesity group (0 vs. 2 components; *p* = 0.050; Cohen’s d = 0.45).

**Figure 2:**
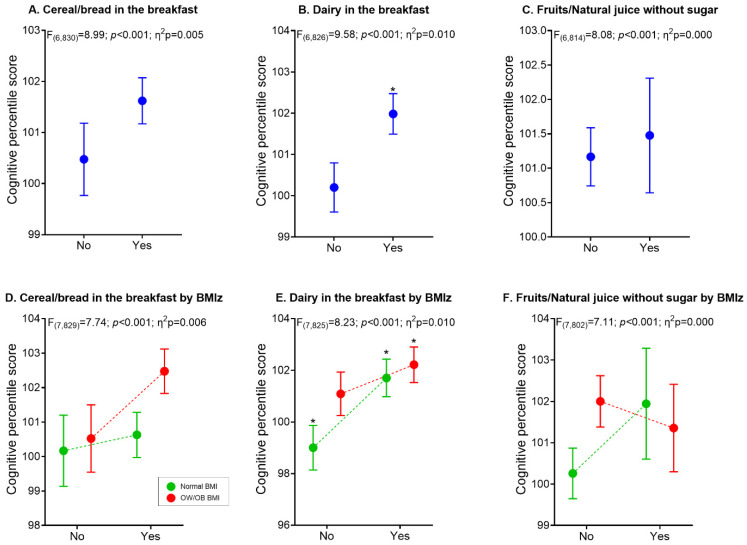
Differences in the adolescents’ cognitive performance according to the three quality breakfast components analyzed. Models in figures (**A**–**C**) were adjusted to sex, peak high velocity, fitness, schools, and body mass index z-score, whereas models in figures (**D**–**F**) were adjusted to sex, peak high velocity, fitness, and schools. * Significative mean differences between groups. BMI: body mass index; OW: overweight; OB: obesity.

Figure 2 shows the three main breakfast quality components associated with all pupils’ cognitive performance (Figure 2A–C) and according to their nutritional status (Figure 2D–F). All six general models were statistically significant (*p* < 0.001). Overall, no differences were found in cereal/bread and fruits/natural juice components between groups (Figure 2A,C). However, adolescents who consumed dairy for breakfast were significantly different from those that did not (*p* = 0.004; Cohen’s d = 0.21) (Figure 2B). No differences in breakfast quality were found in cereal/bread and fruits/natural juice according to BMIz groups (Figure 2D,F). However, differences were observed in the normal-BMIz pupils (0 vs. 2 components; *p* = 0.045; Cohen’s d = 0.46) and the same group (normal-BMIz) compared to the overweight/obesity group (0 vs. 2 components; *p* = 0.047; Cohen’s d = 0.45).

## 4. Discussion

This study aimed to determine whether adolescents who eat breakfast just before a complete cognitive evaluation, do not regularly skip breakfast, and consume a high-quality breakfast present a higher cognitive performance; furthermore, to establish possible differences according to their nutritional status. The main findings were that pupils who had breakfast before a cognitive evaluation, have breakfast regularly, and consume at least two breakfast quality components seem to have greater cognitive performance than those who do not. In this line, higher cognitive performance was present in adolescents who included dairy products. Regarding their nutritional status, cognitive performance was lower in pupils with overweight/obesity and who regularly skipped breakfast compared to those who did not.

Overall, to improve the understanding of how having breakfast influences the brain and, in turn, cognitive performance, it is necessary to consider some relevant factors related to skipping this meal. In this sense, previous studies have shown some mixed and equivocal findings according to the type of breakfast consumed, participants’ characteristics, and the study’s methodological features. Some of these factors are (a) variable analyzed (e.g., energy balance, health parameter, cognitive performance); (b) study population (e.g., athletes, elderly, schoolchildren, socio-economic background); (c) effect of skipping breakfast (as a result of lack of habit) vs. planned intermittent fasting (e.g., skipping breakfast every other day); (d) age, intelligence quotient, physical fitness level, and baseline metabolic characteristics; (e) specificity of breakfast composition; (f) prolonged overnight fast (e.g., last meal); (g) type of participant’s diet (e.g., ketogenic diet); and (h) lack of experimental and well-designed randomized controled trials [39,40,41]. This complex net of factors makes it challenging to establish the real effect and association of breakfast on brain health and compare results.

### 4.1. Skipping Breakfast and Cognitive Performance

In our study, which, methodologically, could be deemed quasi-experimental and ecological research, adolescents having breakfast just before a cognitive demand showed enhanced cognitive performance compared to those who did not. Furthermore, no evidence of differences according to their nutritional status was established. In this sense, several studies showed that skipping breakfast impacts the children and adolescents’ cognitive and academic achievement, negatively impairing their problem-solving ability, attention, and episodic memory in the morning [12,13,42]. However, this detrimental cognitive effect was not observed in children (8–10 years) who regularly consumed breakfast and skipped it once [43]. In the case of children with obesity and “breakfast skippers”, it has been established that they present some metabolic alterations (e.g., leptin gene mutation, reduction of carbohydrate utilisation) associated with a reduction in attention [15] and verbal, non-verbal, and short-term memory [44]. It is essential to highlight that while skipping breakfast has been related to a higher risk of obesity, the current evidence is ambiguous [40].

Despite all these relevant aspects, it is also crucial to consider the adolescents’ social context and familial support, because most “breakfast skippers” have unfavorable socio-economic backgrounds [12,45]. Social factors have been related to worse food quality and physical inactivity, among others, influencing a higher rate of overweight/obesity [46]. This vicious circle, which negatively affects cognition and academic achievement, seems to improve if children and adolescents are educated and receive subsidized breakfast at school [47]. More studies are necessary to have a clear relationship between skipping breakfast, obesity, and cognitive performance.

### 4.2. Breakfast Quality and Cognitive Performance

#### 4.2.1. Cereals and Fruits

It is well known that a high-quality breakfast is a great source of amino acids, vitamins, minerals, antioxidants, and fiber, which positively impacts children’s global health and cognition [5,6,7]. In this way, evidence indicates that breakfast composition may affect behavior, cognition, and learning in children and adolescents [47,48,49,50]. In our study, both cereals and fruits (and fruit juices without added sugar) were not associated with significant cognitive performance differences. However, scientific literature has shown inconclusive results on this matter; for instance, some authors declare that eating cereal as part of the breakfast reduces attention deficit by more than a half, improving immediate word recall [13]. This favorable result has been associated with the breakfast’s glycemic index; however, an experimental study showed improvements in cognitive tasks when adolescents consumed a high glycemic index breakfast [40]. Furthermore, a recent systematic review concluded that a favorable cognitive function is observed independently when having a low or high glycemic index breakfast [51].

On the other hand, fruit consumption has been associated with several beneficial health indicators, such as lower cardiovascular risk, cancer, chronic diseases, and illness [52]. The concentration of flavonoids and antioxidants in fruits have been shown to ameliorate cognitive impairment [53]; however, an experimental study in an animal model showed that fructose consumption reduces hippocampal synaptic plasticity, which underlies cognitive performance [54]. In particular, the protocol study by Cisternas et al. (2015) [54] induced metabolic syndrome in mice consuming fructose (15%) and observed a significant reduction of the hippocampus to sustain synaptic plasticity (number of contact zones and the size of postsynaptic densities) and hippocampal neurogenesis after eight weeks. These findings were confirmed by Jiménez-Maldonado et al. (2018) [55], concluding that a short period of fructose consumption can affect brain plasticity without altering the peripheral metabolic dysfunction. In addition, recently, a theoretical article indicated that fructose can disrupt cerebral metabolism and neuronal function, leading to Alzheimer’s disease [56]. Experimental and longitudinal studies in humans are necessary to understand how fructose can affect the human brain and cognitive functioning.

#### 4.2.2. Dairy Products

Based on epidemiological and experimental studies, a reduced risk of obesity and cardiovascular disease was observed when people included dairy products (e.g., milk, yoghurt, cheese) in their daily diet [57]. Additionally, dairy in breakfast has an important role on improving growth and brain function in school-age children [16]; indeed, fortified milk could be an effective way to obtain polyunsaturated fatty acids, a crucial nutrient favouring brain development [58]. Furthermore, dairy products are rich in micronutrients, such as vitamins (C, D, B6, B12) and minerals (calcium, zinc, selenium) [5,59], all of which are relevant to the brain development and function [60]. Our findings support this conclusion, because adolescents who included dairy in breakfast had greater global cognitive performance results.

On the other side, few children and adolescents include dairy products in their breakfast due to socio-economic and educational factors [61,62]. For instance, a study in Spanish schoolchildren (9–13 years-old) found that 32% of girls and 17% of boys had a dairy product (e.g., milk, yoghurt) for breakfast [63]. In general, dairy products’ consumption is low in diverse countries [62], challenging the dietary calcium recommendations associated with a diet deficient in several nutrients [64]. Finally, calcium consumption has been related to fat oxidation in adults [57,65]. Therefore, it is possible to speculate that dairy products could be deemed a mediator in the relationship between obesity and cognitive performance. Mediation and moderation studies are necessary to elucidate this and other research questions related to this crucial nutritional matter in adolescents’ brain development.

### 4.3. Future Studies, Strengths and Limitations

Some questions emerged from the present findings that may complement this research area in future studies related to adolescents’ cognitive performance, such as (a) the long-term effects of breakfast, (b) the relationship between diverse macro- and micronutrients, (c) the impact of breakfast in children and adolescents with poor nutritional status, and (d) the influence of socio-economic and vulnerability factors.

Finally, our study presented some limitations; for instance, the applied questionnaires did not assess for specific compositions of breakfast or the quantities of each food and nutrients consumed. Additionally, the assessment was done verbally, which is known to be biased. Furthermore, the cross-sectional design precluded the determination of causality, even with careful adjustment for covariates. Finally, the lack of a personal socioeconomic indicator must be considered. Nonetheless, our study had some strengths, such as the large sample size, the inclusion of different cognitive tasks to compute a global cognitive score, and the quality of potential confounders included in our analyses (i.e., a global physical fitness score). To our knowledge, this is the first study that evidences the relationship between breakfast consumption and cognitive performance in adolescents in Latin America.

## 5. Conclusions

Adolescents with a normal-BMIz and those with overweight/obesity had higher performance when having breakfast just before a cognitive evaluation and including at least two breakfast quality components, particularly, dairy products. Skipping breakfast regularly was negatively linked with lower cognitive performance, especially in adolescents living with overweight/obesity. These findings suggest that parents and children’s nutritional education at school and on a public health level, regarding the importance of having breakfast, could be an influent strategy for improving cognitive performance. Intervention studies are needed to corroborate the present results.

## Figures and Tables

**Table 1:** Adolescents’ characteristics according to nutritional status.

Variables	Overall	Normal BMI	OW/OB BMI	*p*-Value
	(*n* = 1181)	(*n* = 563)	(*n* = 618)	
Age (years)	11.7 ± 1.06	11.8 ± 1.1	11.6 ± 1.1	<0.001
Sex (girls/boys)	580/601	280/283	300/318	0.683
Weight (kg)	50.3 ± 11.9	42.9 ± 7.6	57.1 ± 11.0	<0.001
Height (cm)	152.4 ± 9.2	151.8 ± 9.6	153.1 ± 8.8	<0.001
BMIz-score	1.04 ± 1.07	0.13 ± 0.6	1.88 ± 0.6	<0.001
Cognitive test score	100.0 ± 8.8	100.5 ± 8.8	99.6 ± 8.9	<0.001
Having breakfast before a cognitive test				0.496
Yes	693 (67.5%)	329 (32.0%)	364 (35.4%)	
No	334 (32.5%)	151(14.7%)	183 (17.9%)	
Skipping breakfast regularly				<0.001
Yes	247 (24.0%)	96 (9.5%)	151 (14.6%)	
No	782 (76.0%)	399 (38.7%)	383 (37.2%)	
Breakfast quality score				0.570
0 point	171 (17.2%)	76 (7.6%)	95 (9.6%)	
1 point	319 (32.1%)	159 (16.0%)	160 (16.1%)	
2 points	372 (37.4%)	176 (17.8%)	196 (19.7%)	
3 points	132 (13.3%)	68 (6.8%)	64 (6.4%)	
Fruits/natural juice without sugar				0.382
Yes	199 (19.9%)	90 (9.0%)	109 (10.9%)	
No	801 (80.1%)	390 (39.0%)	411 (41.1%)	
Cereals/bread in the breakfast				0.506
Yes	726 (69.7%)	354 (34.0%)	372 (35.7%)	
No	316 (30.3%)	147 (14.1%)	169 (16.2%)	
Dairy in the breakfast				0.188
Yes	622 (60.2%)	309 (30.0%)	313 (30.3%)	
No	411 (39.8%)	187 (18.1%)	224 (21.6%)	

Values are presented as means and standard deviation (±) or frequencies and percentages (%), *t*-student or Chi-square tests for comparisons between groups (*p* < 0.05); BMI: body mass index; OW: overweight; OB; obesity.

## Data Availability

The data presented in this study are available on request from the corresponding author. The data are not publicly available due to ethical concerns.
